# Sequence-dependent electrical response of ssDNA-decorated carbon nanotube, field-effect transistors to dopamine

**DOI:** 10.3762/bjnano.5.220

**Published:** 2014-11-13

**Authors:** Hari Krishna Salila Vijayalal Mohan, Jianing An, Lianxi Zheng

**Affiliations:** 1 School of Mechanical and Aerospace Engineering, Nanyang Technological University, 50 Nanyang Avenue, 639798, Singapore,; 2 Department of Mechanical Engineering, Khalifa University of Science, Technology & Research (KUSTAR), P.O. Box 127788, Abu Dhabi, United Arab Emirates

**Keywords:** carbon nanotube, deoxyribonucleic acid, dopamine, field-effect transistor, uric acid

## Abstract

Single-walled carbon nanotube (SWCNT)-based field-effect transistors (FETs) have been explored for use as biological/chemical sensors. Dopamine (DA) is a biomolecule with great clinical significance for disease diagnosis, however, SWCNT FETs lack responsivity and selectivity for its detection due to the presence of interfering compounds such as uric acid (UA). Surface modification of CNTs using single-stranded deoxyribonucleic acid (ssDNA) renders the surface responsive to DA and screens the interferent*.* Due to the presence of different bases in ssDNA, it is necessary to investigate the effect of sequence on the FET-based molecular recognition of DA. SWCNT FETs were decorated with homo- and repeated-base ssDNA sequences, and the electrical response induced by DA in the presence and absence of UA was gauged in terms of the variation in transistor electrical parameters including conductance, transconductance, threshold voltage and hysteresis gap. Our results showed that the response of ssDNA-decorated devices to DA, irrespective of the presence or absence of UA, was DNA sequence dependent and exhibited the trend: G > A > C and GA > GT > AC > CT, for homo- and repeated-base sequences, respectively. The different response of various SWCNT–ssDNA systems to DA underlines the sequence selectivity, whereas the detection of DA in the presence of UA highlights the molecular selectivity of the ssDNA-decorated devices.

## Introduction

Single-walled carbon nanotubes (SWCNTs) are excellent chemical/biological sensing materials because of their ultra-high sensitivity, fast response, and size compatibility, as compared to traditional sensors [1–3]. Of the numerous biomolecules, detection of dopamine (DA) is critical because of its high clinical importance in various brain functions such as learning, memory formation, message transfer in the central nervous system and understanding the pathological processes of Parkinson’s disease [4]. However, the presence of interfering compounds such as ascorbic acid (AA) and uric acid (UA) is a major cause for poor response and selectivity in SWCNT-based sensors in the detection of DA. Moreover, current electrochemical methods for CNT-based DA detection suffer from low sensitivity [5–7]. The use of an electronic detection technique with a chemically modified CNT surface that recognizes DA and selectively screens the interferent is a potential solution to overcome these hurdles.

Surface modification improves the interaction strength between the nanotube and DA, thereby enhancing the affinity and specificity of molecular recognition, whereas an electronic route promises faster detection [8–9]. In particular, single-stranded deoxyribonucleic acid (ssDNA) decoration on SWCNT has garnered tremendous attention because of its selectivity and sensitivity towards a wide range of analytes such as ligands, hormones, proteins, enzymes, and vapor-phase odorants, in addition to being economical and readily available [10–11]. ssDNA is a biopolymer composed of a deoxyribose sugar, a phosphate and one or more of the four nitrogenous bases, namely, adenine (A), guanine (G), cytosine (C), or thymine (T), which binds on the SWCNT surface through non-covalent π–π stacking interactions [12]. Moreover, this non-covalent functionalization is more desirable than covalent functionalization methods because it preserves the electronic properties of SWCNT while covalent methods may disrupt the nanotube surface. The presence of multiple bases allows the design of sequences to achieve affinity to different biological/chemical molecules [13]. Thus, ssDNA decoration on SWCNTs achieves the two-fold goal of surface functionalization and receptor immobilization as it renders the CNT surface responsive and acts as a receptor capable of specific and selective binding with the target analyte.

ssDNA-decorated, individually semiconducting SWCNTs in a field-effect transistor (FET) configuration merge the molecular recognition diversity of ssDNA with the excellent electronic properties of SWCNT to provide a fast electronic platform for biosensing [14]. Previously, numerous works have demonstrated that nucleic acid-functionalized CNT-based FETs exhibit fast, specific, and reproducible response to the detection of various polar molecules [15–16]. Since ssDNA has four bases, it gives the possibility of numerous sequence combinations, which interact differently with CNTs as well as DA, and consequently, this influences the FET response. The transistor electrical parameters such as conductance, transconductance, threshold voltage and hysteresis gap extracted from the current–voltage characteristics are indicators of the contributing sensing mechanisms [17], which aid in interpreting the sequence-dependent FET response. Therefore, obtaining this information about the variation in these transistor parameters due to ssDNA–DA interaction will facilitate the design of ssDNA sequences in the development of ssDNA-decorated, SWCNT-based FETs for sensitive and selective detection of DA.

This work is divided into two parts: First, we analyze the effect of ssDNA decoration of SWCNT on the FET response to DA, UA and DA–UA solution mixtures. Second, we interpret the sequence-dependent electrical response of both homo- and repeated-ssDNA-decorated SWCNT FETs to DA, in the presence and absence of UA.

## Experimental

### SWCNT growth and FET fabrication

**Figure 1:**
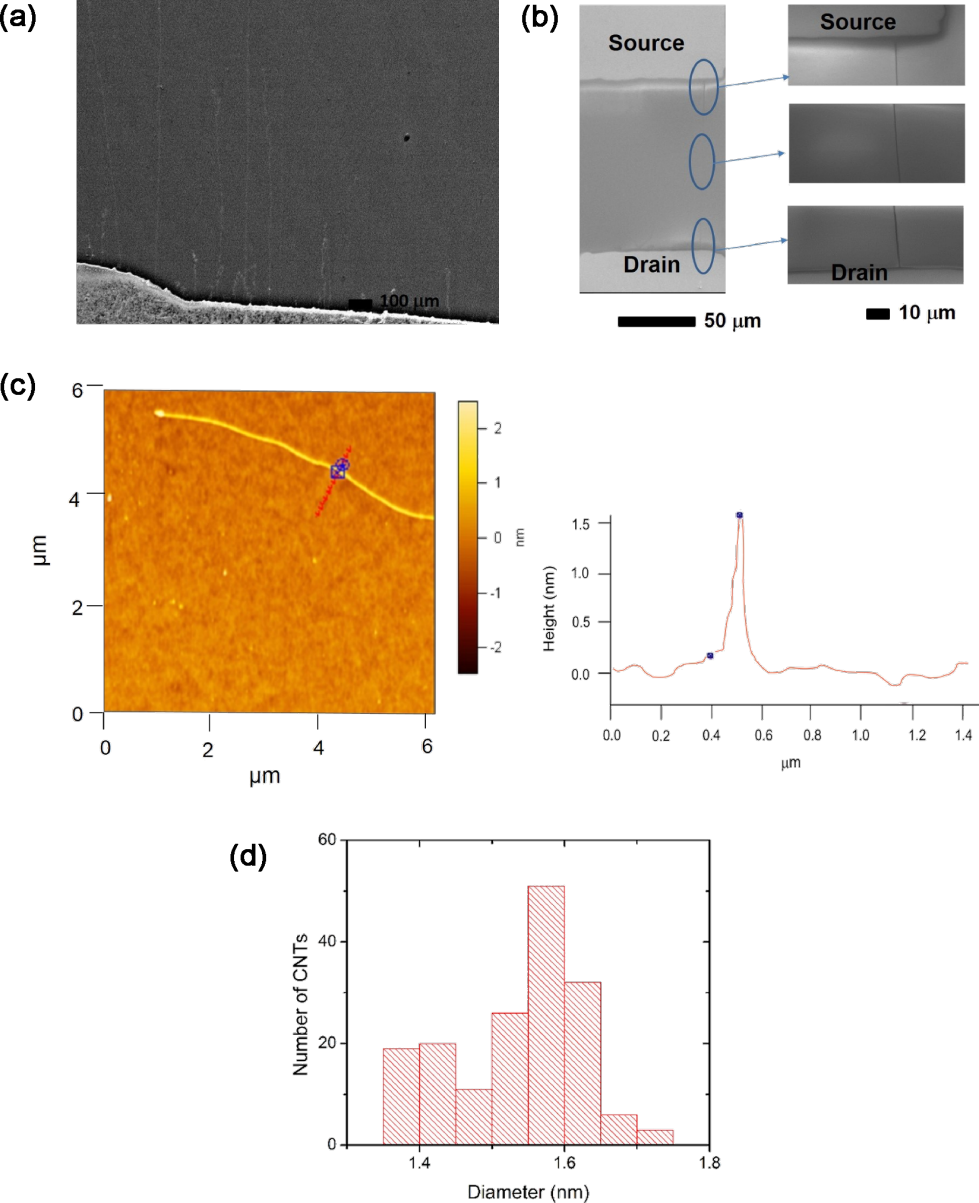
FE-SEM image of (a) CVD grown SWCNTs and (b) a SWCNT FET with an enlarged view of the SWCNT near the source, central and drain regions. (c) AFM image of a single SWCNT and its height profile indicating the SWCNT diameter is ≈1.51 nm. (d) Histogram plot showing the diameter distribution of the SWCNTs. The average diameter of the SWCNTs was ≈1.53 nm.

Long, individual SWCNTs were grown on n^+^-doped Si capped by 1 µm of SiO_2,_ thermally grown via chemical vapor deposition (CVD) using 0.01 M FeCl_3_ ethanol solution as the catalytic precursor, which is similar to our previous works [18–22]. The substrates containing SWCNTs were subjected to shadow mask-facilitated, electron beam evaporation of Ti/Au (5 nm/50 nm) in order to deposit the source and drain electrodes spaced ≈100 μm apart. Figure 1a,b shows the field-emission scanning electron microscopy (FE-SEM) image of the as-grown SWCNT array and a fabricated FET containing a single SWCNT connecting the source and drain electrodes. Figure 1c,d shows the atomic force microscopy (AFM) image of a single SWCNT with its height profile, and the diameter distribution of SWCNTs, respectively.

### ssDNA immobilization

**Figure 2:**
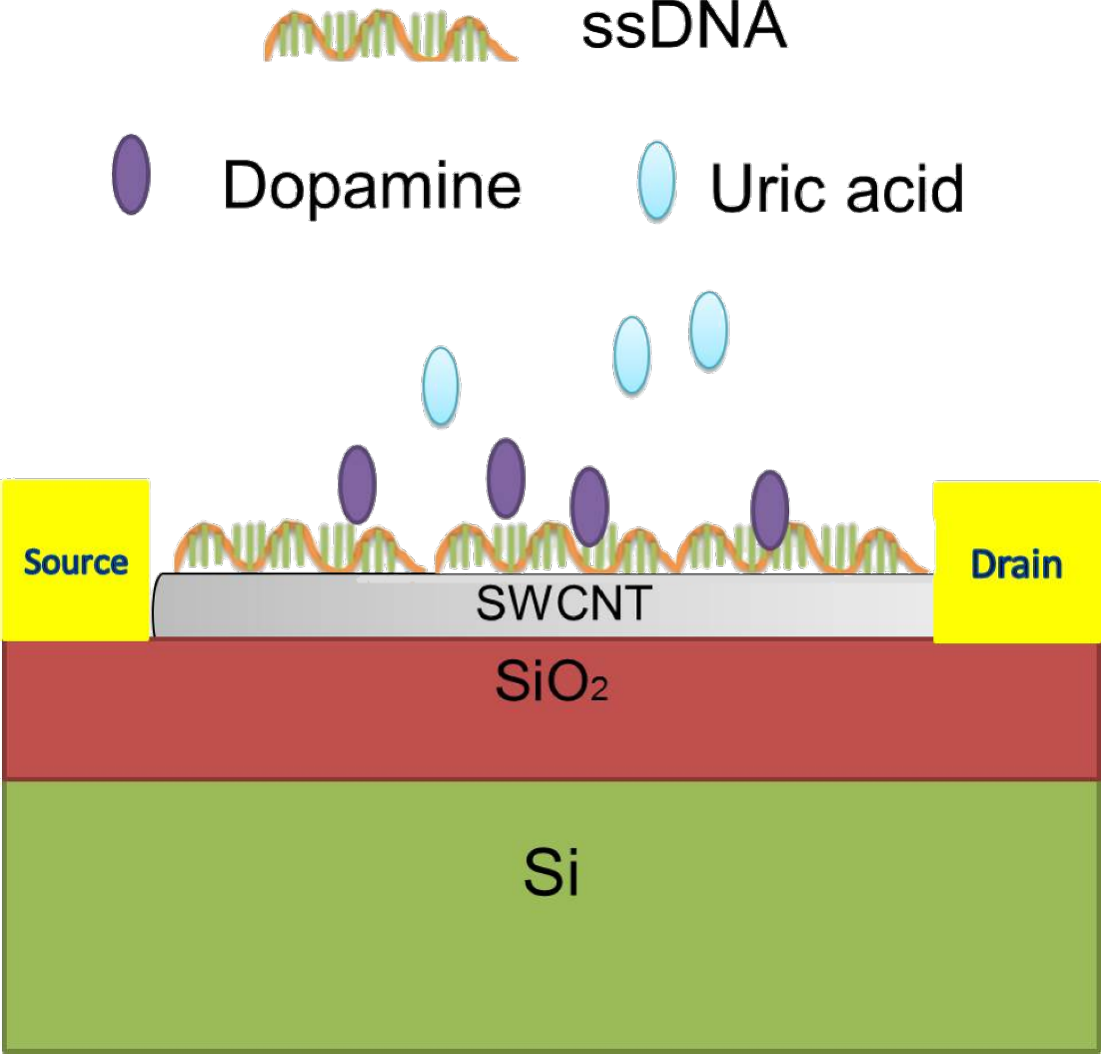
Schematic diagram of ssDNA-decorated SWCNT-based FET for DA detection.

SWCNTs from a single CVD growth run were used to avoid device variability. Eight ssDNA sequences (G_22_, A_22_, C_22_, T_22_, (GT)_22_, (GA)_22_, (AC)_22_, and (CT)_22_) of the same sequence length, dopamine 3-hydroxytyramine, and uric acid were purchased from Sigma-Aldrich, Singapore. Repeating base ssDNA sequences (AT)_22_ and (GC)_22_ were not used in this study because these sequences have high self-complementarity resulting in the formation of undesirable aggregates. All solutions were prepared using phosphate buffer saline (PBS, pH 7.4, BST Scientific, Ltd.) unless otherwise stated. For ssDNA immobilization, a 5 µL drop of 10 µM ssDNA was pipetted onto the devices and incubated in a 100% humid environment for about 1 h, and then the drop was removed by blowing nitrogen gas. Thereafter, 5 µL of 1 µM of either DA, or UA, or DA–UA solution mixture (1:1), was pipetted onto the devices, which were decorated with different ssDNA sequences. Subsequently, the devices were incubated for 15–20 minutes in a humid environment, rinsed with PBS and deionized (DI) water, and dried by blowing nitrogen gas. Figure 2 shows the schematic setup of a typical ssDNA-decorated SWCNT-based FET for DA sensing.

### Electrical characterization

All *I*–*V* measurements were made using a semiconductor device analyzer (Agilent, 4156B). The drain current (*I*_D_) versus gate voltage (*V*_G_) characteristics (transfer) were obtained at a drain voltage (*V*_DS_) of 1 V. The on conductance (*G*_on_) is obtained from the slope of the *I*_D_*–V_DS_* (output) curve between −0.1 V to 0.1 V at a constant *V*_G_ of −24 V corresponding to the on state of the FET. The threshold voltage (*V*_th_) is the voltage demarcating the on and off states. It is obtained by extrapolating the steepest portion of the transfer curves to intersect the *x*-axis for both forward and reverse gate voltage sweeps. The hysteresis gap (*H*) is the difference in threshold voltage between the forward and reverse sweep threshold voltage. The slope of the *I*_D_*–V*_G_ plot near the threshold voltage gives the peak transconductance (*g*_mp_).

A total of 168 devices out of more than 500 fabricated devices with a single SWCNT as a channel and a current on/off ratio of >1000 were selected to ensure the semiconducting nature of the devices. ssDNA decoration resulted in a 15.6 ± 5.9% drop in *G*_on_, a 14.8 ± 4.9% drop in *g*_mp_, a –4.1 ± 0.5 V negative shift in *V*_th_ and a 0.9 ± 0.7 V change in *H.* Roughly, for each ssDNA sequence, 20 devices were used: 8 for DA, 5 for UA and 7 devices for a solution mixture of DA and UA.

## Results and Discussion

### Effect of ssDNA decoration on SWCNT FET response

**Figure 3:**
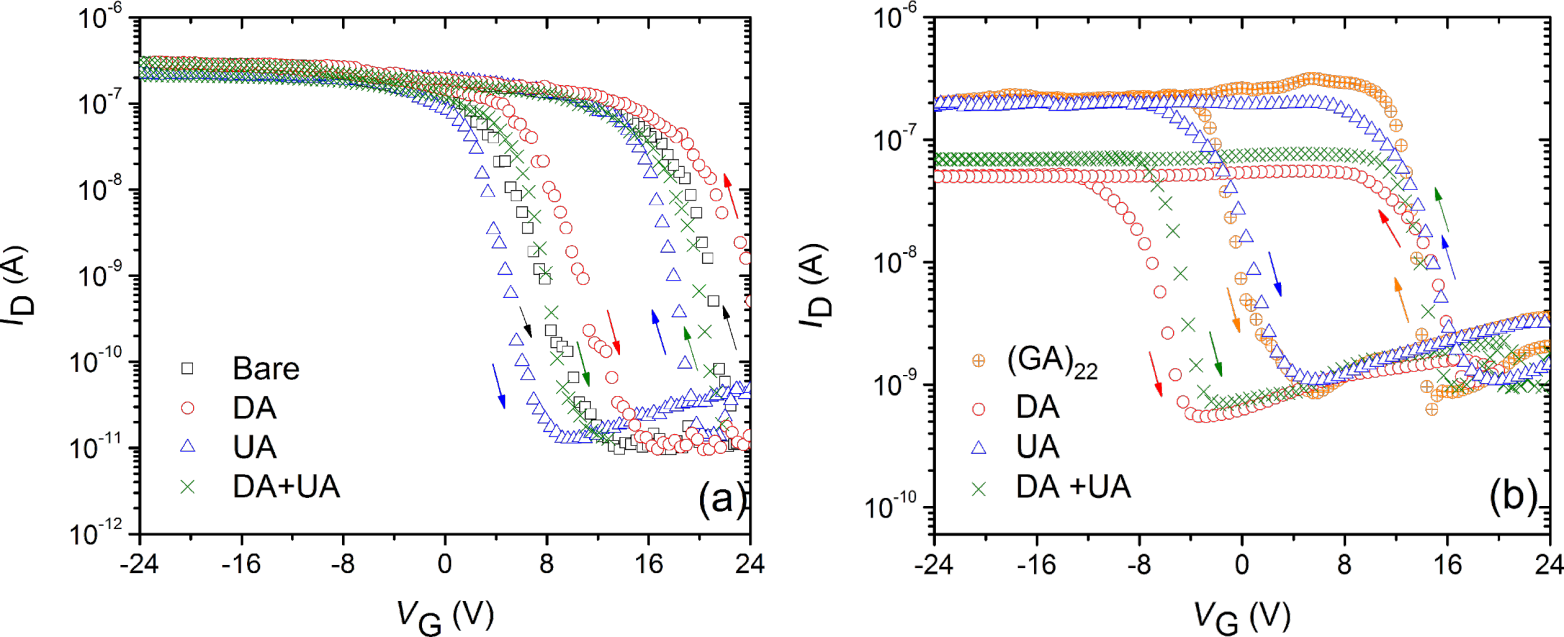
*I*_D_*–V*_G_ curve of (a) bare SWCNT FET and (b) (GA)_22_-decorated SWCNT FET exposed to DA, UA and a DA–UA solution mixture. The arrows indicate the direction of the gate voltage sweep.

The FETs fabricated from bare SWCNT and DNA-modified SWCNT were tested with DA, UA and a solution mixture of both, and the typical transfer characteristics are shown in Figure 3. According Figure 3a, FETs with bare SWCNT after exposure to DA displayed a slight positive shift in *V*_th_ (about +0.8 V) whereas UA exposure showed a negative shift in *V*_th_ (about −0.6 V), However, neither of them produced any significant change in *G*_on_ (<5%), or *g*_mp_ (<5%), or *H* (<1 V). Especially, a solution mixture of DA and UA failed to produce any effect on the transfer curves.

DA carries a positive charge in PBS at pH 7.4, while UA has a negative charge in PBS at the same pH [23]. Thus, the observed opposite directional shift in the transfer curves for DA and UA is attributable to the slight electron withdrawal from the SWCNT by cationic DA and electron donation to the SWCNT by anionic UA [24–25]. However, the simultaneous presence of opposite charges in a solution mixture of DA and UA stabilizes one another through electrostatic forces; thereby the neutral mixture fails to produce any effect on the SWCNT [23]. Thus, detecting DA in the presence of UA is a challenge because of the electrical neutrality of the mixture and the hydrophobic nature of the SWCNT. This discourages the attachment of polar molecules onto the surface, and results in a non-reliable and non-reproducible charge transfer between DA/UA and the SWCNT.

Figure 3b shows the transfer curves of a SWCNT FET decorated with (GA)_22_ sequence tested with DA, UA, and DA–UA solution mixtures. Upon exposure to DA, the (GA)_22_-decorated device displayed a reduction in *G*_on_ (≈75.8%), decrease in *g*_mp_ (≈81.1%), negative shift in *V*_th_ (≈−6 V), and an increase in *H* (≈8 V). A device coated with the same sequence, tested with UA, yielded no response. More importantly, exposure of the (GA)_22_-decorated FET to the DA–UA solution mixture produced the same effect on the transfer curve as seen when exposed to DA alone, however, with a slightly lower magnitude of change in transistor parameters. Namely, a reduction in *G*_on_ (≈67.1%), decrease in *g*_mp_ (≈74.2%), negative shift in *V*_th_ (≈−4.8 V), and increase in *H* (≈6 V) was observed. Comparing Figure 3a and Figure 3b, it was found that cationic DA as well as the DA–UA solution mixture produced a negative *V*_th_ shift in the (GA)_22_-decorated FET. This is in contrast to the *I*–*V* response of bare SWCNT FET to DA, suggesting the interaction between ssDNA and DA.

The reduction in *G*_on_ and *g*_mp_, the negative shift in *V*_th_, and the increase in *H* indicates the contribution of carrier scattering, charge transfer and charge trapping mechanisms, respectively [17]. From the above results, three important points are noteworthy. First, the absence of the response to UA in the ssDNA-decorated device suggests the lack of interaction between ssDNA and UA. Second, the similar effect of the DA and DA–UA solution mixtures on the transfer curve of ssDNA-coated FETs confirms that response is due to the interaction between ssDNA and DA. Third, compared to bare SWCNT FETs, the change in magnitude of the transistor parameters are much higher in ssDNA-decorated FETs, even in the presence of UA. This highlights the enhancement in device response by ssDNA surface modification, and the improvement in selectivity of DA recognition in the presence of UA.

To interpret the influence of ssDNA surface modification on the response of SWCNT FETs to DA, UA and DA–UA solution mixtures, the nature of the SWCNT–ssDNA interaction requires attention [26–27]. In general, all the ssDNA-decorated devices exhibited a left shift in *V*_th_ by ≈5 V (because of electron donation by the negatively charged ssDNA), and a reduced *G*_on_ by about 15–35% compared to bare devices, suggesting carrier scattering by molecular coating [16]. There were no major changes observed for *g*_mp_ or *H*. According to molecular dynamics (MD) simulations [28–29], ssDNA interacts with the SWCNT sidewall through its DNA bases [30–31], with a significant number of the bases being desorbed [32]. The hydrophobic nature of the nitrogenous bases of the ssDNA and SWCNT surface results in high affinity between them, however, the hydrophilic phosphate groups of ssDNA do not favor the nanotube surface. Adsorption of the ssDNA on the SWCNT results in a hydrophilic environment around the nanotube because of the negatively charged phosphate backbone of ssDNA. Moreover, this hydrophilicity further increases as ssDNA binds more to the SWCNT. Thus, ssDNA decoration causes successful conversion of the hydrophobic SWCNT surface to hydrophilic, thereby enhancing the possibility of formation of a set of binding pockets within the proximity of the SWCNT sidewall. This hydrophilic environment on the SWCNT surface attracts polar analytes onto these hydration layers and solvates them, resulting in an increasing binding affinity of the polar analytes to these pockets. As more polar molecules are adsorbed, the electrostatic potential around the SWCNT is modulated. Thus, the conversion of hydrophobic SWCNT to hydrophilic provides a congenial environment for the ssDNA–DA interaction on the SWCNT surface, which is responsible for the observed improvement in device response.

### Mechanism of FET-based ssDNA–DA interaction detection

The plausible cause of the observed response of the ssDNA-decorated device to DA, UA and DA–UA mixtures (i.e., the the change of the sign in the electrical parameters (−*∆G*_on_/*G*_on_, −*∆g*_mp_/*g*_mp_, *−∆V*_th_ and *+∆H*), is interpreted based on the ssDNA–DA/UA interaction [26–27]. The binding of ssDNA with DA could be because of strong interactions such as hydrogen bonding, or mutual association of the hydrophobic regions between DA and ssDNA, or weak electrostatic attractions. According to earlier reports [33], the electrostatic forces are the dominant forces of interaction between ssDNA and DA. Since DA is positively charged, it is attracted towards the ssDNA main chain resulting in strong electrostatic attractive forces between ssDNA phosphate group and DA. Moreover, the two –OH groups in DA possess hydrogen donor/acceptor ability, and hence, compete with the positively charged region of DA for the ssDNA phosphate groups. Consequently, the positively charged 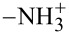 groups of DA are rendered free, thereby enabling them to interact with DNA bases. This allows DA to participate in the solvation mechanism of DNA bases through hydrogen bonding interactions. Thus, the 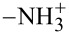
group of DA is protonated during solvation by the DNA hydration layer, which is reflected as the decline in channel conductance (−*∆G*_on_/*G*_on_) [34]. The DA protonation results in electron transfer from the DNA to the SWCNT causing a change in the electrostatic environment around the nanotube, which is responsible for the observed shift in threshold voltage (−*∆V*_th_) [25]. The electron donation to the SWCNT reduces the charge carrier concentration inside the nanotubes resulting in the formation of static charges, which reduces the slope of the transfer curve by generation of carrier scattering (*−∆g*_mp_/*g*_mp_) [17]. Furthermore, the charge trapping ability of the ssDNA–DA complex containing bases G and A, which have better affinity to SWCNT [35], causes the charge traps formed by ssDNA–DA adducts to be in greater proximity to the nanotube surface, thus, increasing the hysteresis gap (*+∆H*).

Anions such as UA are predominantly involved in hydrogen bond interactions with C, A and G bases [23]. Moreover, the repulsion between the negatively charged phosphate backbone of the ssDNA and the negatively charged UA will screen UA from interacting with the ssDNA, resulting in the lack of response in FETs exposed to UA alone. Moreover, it is clear that exposure of the ssDNA-decorated device to the DA–UA mixture had the same effect on the transfer characteristics as shown by devices exposed only to DA, although, with lower magnitude. This is attributable to the reduction in the proportion of DA molecules interacting with ssDNA due to its electrostatic attraction to negatively charged UA molecules.

### ssDNA sequence-dependent FET response

**Figure 4:**
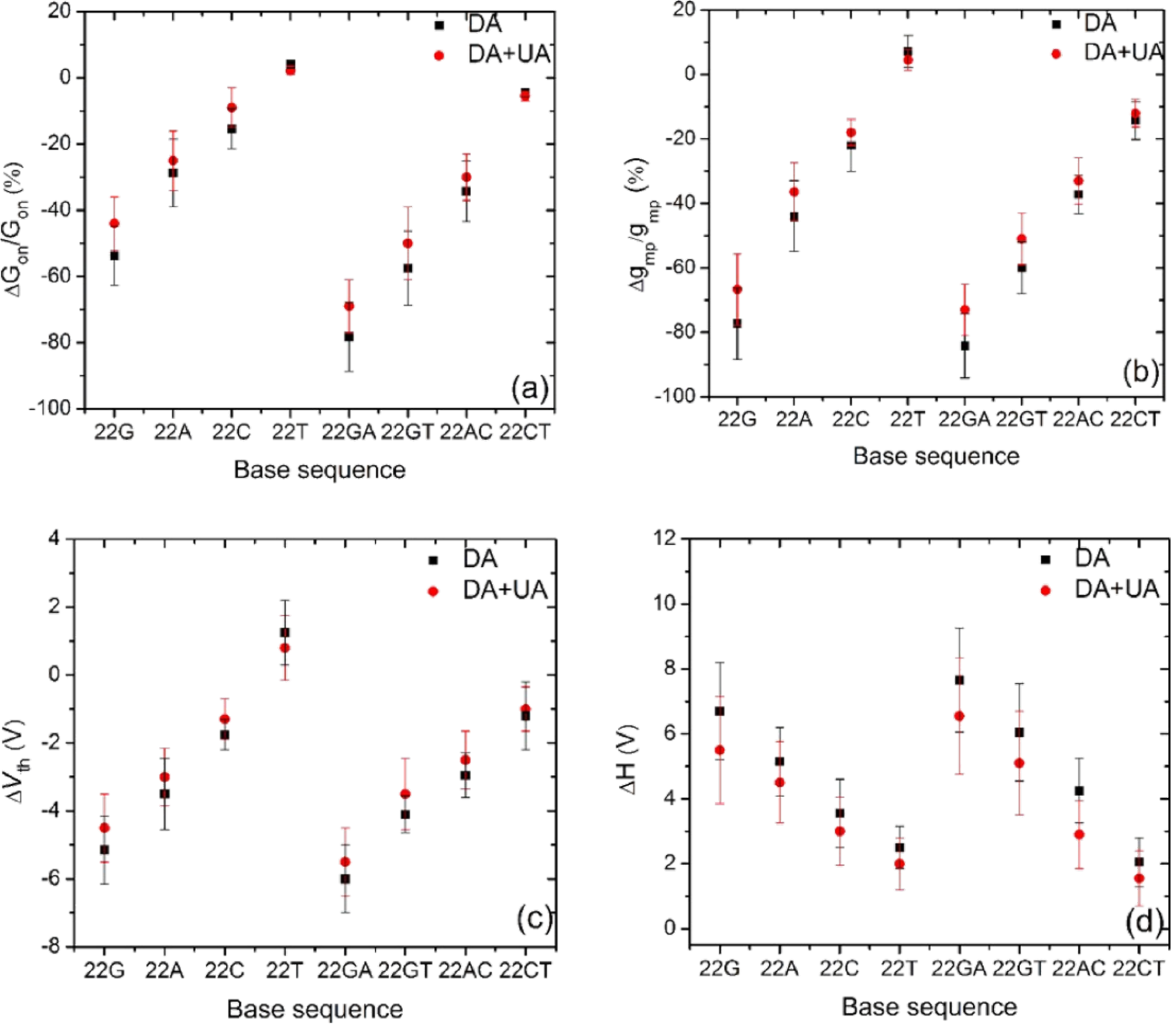
Summary of the sequence-dependent variation in transistor electrical parameters induced by the DA and DA–UA solution mixtures. (a) *∆G*_on_/*G*_on_, (b) *∆g*_mp_/*g*_mp_, (c) *∆V*_th_, and (d) *∆H*. The “−“ sign on the *y*-axis in (a) and (b) indicates the decrease in the respective parameters with respect to the ssDNA-functionalized state. The “−“ and “+” on the *y*-axis in (c) and (d) indicate the left shift in *V*_th_ and increase in *H*, respectively, with respect to the ssDNA functionalized state.

The binding pockets created by ssDNA on SWCNT are sequence dependent, and consequently, the ssDNA–DA interaction leads to a sequence-dependent FET response. Since many combinations of the base sequence are possible, we investigate the effect of sequence alteration on the device response. Figure 4 summarizes the relative change in the on conductance (*∆G*_on_/*G*_on_), the change in the peak transconductance *(∆g*_mp_/*g*_mp_), the shift in the threshold voltage (∆*V*_th_) and the change in the hysteresis gap (*∆H*) for homo- and repeated-base sequences due to the ssDNA–DA interaction in the presence and absence of UA.

For the homo sequences G_22_, A_22,_ and C_22_, following DA interaction, *G*_on_ dropped by about 55.2%, 29.4% and 18.4% whereas *g*_mp_ decreased by about 80.6%, 46.4% and 21.3%, respectively (Figure 4a,b). For the same order of sequences, ∆*V*_th_ shifted by about −5.1 V, −3.8 V, and −1.8 V, respectively (Figure 4c). On the other hand, *H* increased by about 6.9 V, 4.7 V and 3.5 V for the sequences G_22_, A_22,_ and C_22_, respectively (Figure 4d). For repeated-base sequences (GA)_22_, (GT)_22_, (AC)_22_ and (CT)_22_, *G*_on_ reduced by about 80.3%, 62.2%, 39.5% and 5.1%, and *g*_mp_ decreased by about 87.4%, 63.2%, 34.2% and 10.2%, respectively (Figure 4a and 4b). For the same order of sequences, *V*_th_ displayed a negative shift of about −6.1 V, −3.9 V, −3.1 V, and −1 V, respectively (Figure 4c). On the other hand, *H* increased by about 7.4 V, 6.1 V, 4.1 V and 1.3 V, for sequences (GA)_22_, (GT)_22_, (AC)_22_ and (CT)_22_, respectively (Figure 4d).

To confirm that the ssDNA sequences decorated on SWCNT FETs can selectively identify DA, a solution mixture of DA–UA was used. From Figure 4a–d, clearly, the DA–UA mixture produced the same trend in the FET response as displayed by devices exposed to DA alone, but with a lower magnitude response, for the reason stated earlier. The DA interaction with the ssDNA-decorated SWCNTs elicited a base-dependent trend as follows: G_22_ > A_22_ > C_22_ and (GA)_22_ > (GT)_22_ > (AC)_22_ > (CT)_22_ for homo- and repeated-base sequences, respectively. Since devices decorated with T_22_ showed no significant change in *G*_on_ and *g*_mp_, and an ≈1 V right shift in *V*_th_ (similar to the transfer curve of bare SWCNT exposed to DA (Figure 3a)), it has been omitted from the above trend.

The magnitude of the change in transistor electrical parameters induced by DA is determined by the strength and nature of the SWCNT–ssDNA and ssDNA–DA interactions [25–26]. The observed trend in the change of magnitude in transistor parameters could be addressed based on the contributions from the differences in the binding affinity, wrapping tendency and solvation effects for different bases [36–37]. The binding affinity and wrapping tendency of the different bases on the SWCNT surface follow a particular trend [26,37]. The base-SWCNT binding free energies (*∆E*_bind_) based on thermodynamic integration follows the trend given by: [38–40]

[1]
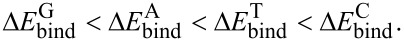


The total binding energy of ssDNA on SWCNT will approximately scale with the sum of the individual base binding free energies as:

[2]
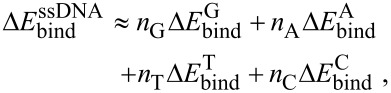


where *n*_A_, *n*_C_, *n*_G_, and *n*_T_ are the number of A, C, G and T in the sequence, respectively [38–40].

On the basis of the above trend, the homo- and repeated-base ssDNAs are expected to display the following trends for binding affinity with SWCNT: G_22_ > A_22_ > T_22_ > C_22_ and (GA)_22_ > (GT)_22_ > (AC)_22_ > (CT)_22_. Clearly_,_ the devices with the sequences G_22_ and (GA)_22_ displayed the best response to DA in the presence or absence of UA. This is similar to the trend observed in our results for DA detection, except for the absence of T_22_. Thus, the strength of SWCNT–ssDNA interaction determines the magnitude of the electrical response to the DA and DA–UA mixtures. In addition, the presence of fewer hydrogen donor/acceptor sites in bases C and T could also contribute to the lower magnitude response of transistor parameters for DA detection compared to bases G and A [41]. Another possibility capable of enhancing the response of ssDNA-containing bases G and A to DA is the unbound interaction known as the edge-to-face NH–π interaction [37,42]. This occurs between the hydrogen of the amino groups of these bases facing the aromatic ring system of DA. Compared to other sequences, T_22_-decorated devices showed an opposite directional shift in *V*_th_ following DA or DA–UA exposure. According to previous reports [37,43], ssDNA with a high pyrimidine content (G and A) has a very good wrapping tendency, as compared to ssDNA with a higher purine content (C and T). Consequently, major portions of the SWCNT are left exposed following the helical wrapping of T_22_, thereby allowing DA to directly interact with the SWCNT surface. Even if DA interacts with the T_22_-decorated SWCNT, the low affinity of T_22_ on the SWCNT will fail to produce any significant observable response.

Thus, different SWCNT–ssDNA systems exhibit a different magnitude response to the DA and DA–UA mixtures, which is a good indicator of the sequence-dependent discriminating capacity of the FETs.

## Conclusion

In conclusion, the surface modification of SWCNTs using ssDNA improved the response of the SWCNT FET to DA, which otherwise showed poor response. The electrical response of a ssDNA-decorated SWCNT FET to dopamine was characterized by reduced conductance and transconductance, a left shift in threshold voltage, and an increased hysteresis gap, which indicates the combination of carrier scattering, charge transfer and charge trapping mechanisms. In addition, ssDNA decoration of the SWCNT improved DA recognition even in the presence of the interferent, UA, which highlights the enhancement in molecular selectivity. The devices exhibited a sequence-dependent trend with sequences G_22_ and (GA)_22_ with the best response to dopamine. Furthermore, the sequence-dependent response of ssDNA-decorated SWCNT FETs to DA highlights the discriminating ability of the different SWCNT–ssDNA systems in recognizing different ssDNA–DA interactions.
